# CNN-Based Cross-Modality Fusion for Enhanced Breast Cancer Detection Using Mammography and Ultrasound

**DOI:** 10.3390/tomography10120145

**Published:** 2024-12-12

**Authors:** Yi-Ming Wang, Chi-Yuan Wang, Kuo-Ying Liu, Yung-Hui Huang, Tai-Been Chen, Kon-Ning Chiu, Chih-Yu Liang, Nan-Han Lu

**Affiliations:** 1 Department of Critical Care Medicine, E-DA Hospital, I-Shou University, Kaohsiung City 824005, Taiwan; ed101632@edah.org.tw; 2 Department of Medical Imaging and Radiological Science, I-Shou University, Kaohsiung City 824005, Taiwan; wang1b011@isu.edu.tw (C.-Y.W.); yhhuang@isu.edu.tw (Y.-H.H.); 3 Department of Radiology, E-DA Cancer Hospital, I-Shou University, Kaohsiung City 824005, Taiwan; ed102500@edah.org.tw; 4 Department of Radiological Technology, Faculty of Medical Technology, Teikyo University, Tokyo 173-8605, Japan; chen.tb@gmail.com; 5 Department of Business Management, National Sun Yat-sen University, Kaohsiung City 804201, Taiwan; chiutony@yahoo.com; 6 Institute of Medical Science and Technology, National Sun Yat-sen University, Kaohsiung City 804201, Taiwan; 7 Department of Emergency Medicine, E-DA Hospital, I-Shou University, Kaohsiung City 824005, Taiwan; 8 School of Medicine, College of Medicine, I-Shou University, Kaohsiung City 824005, Taiwan

**Keywords:** artificial intelligence, deep learning algorithms, convolutional neural networks, breast cancer

## Abstract

**Background/Objectives:** Breast cancer is a leading cause of mortality among women in Taiwan and globally. Non-invasive imaging methods, such as mammography and ultrasound, are critical for early detection, yet standalone modalities have limitations in regard to their diagnostic accuracy. This study aims to enhance breast cancer detection through a cross-modality fusion approach combining mammography and ultrasound imaging, using advanced convolutional neural network (CNN) architectures. **Materials and Methods:** Breast images were sourced from public datasets, including the RSNA, the PAS, and Kaggle, and categorized into malignant and benign groups. Data augmentation techniques were used to address imbalances in the ultrasound dataset. Three models were developed: (1) pre-trained CNNs integrated with machine learning classifiers, (2) transfer learning-based CNNs, and (3) a custom-designed 17-layer CNN for direct classification. The performance of the models was evaluated using metrics such as accuracy and the Kappa score. **Results:** The custom 17-layer CNN outperformed the other models, achieving an accuracy of 0.964 and a Kappa score of 0.927. The transfer learning model achieved moderate performance (accuracy 0.846, Kappa 0.694), while the pre-trained CNNs with machine learning classifiers yielded the lowest results (accuracy 0.780, Kappa 0.559). Cross-modality fusion proved effective in leveraging the complementary strengths of mammography and ultrasound imaging. **Conclusions:** This study demonstrates the potential of cross-modality imaging and tailored CNN architectures to significantly improve diagnostic accuracy and reliability in breast cancer detection. The custom-designed model offers a practical solution for early detection, potentially reducing false positives and false negatives, and improving patient outcomes through timely and accurate diagnosis.

## 1. Introduction

Breast cancer remains one of the most significant health challenges worldwide, particularly among women [1]. In 2021, breast cancer was the most diagnosed cancer among women in Taiwan, with an age-adjusted incidence rate of 82.51 per 100,000 women. It also ranked as the fourth leading cause of cancer-related deaths among Taiwanese women, with an age-adjusted mortality rate of 13.77 per 100,000 women. The incidence of breast cancer in Taiwan has been increasing over the past few decades, reflecting changes in lifestyle, reproductive patterns, and increased awareness leading to more screenings. The peak age of breast cancer diagnosis in Taiwan falls between 45 and 69 years, indicating a significant impact on women in their most productive years.

The importance of non-invasive imaging techniques, such as mammography and ultrasound, in the early detection of breast cancer cannot be overstated [2,3,4]. These methods have been widely adopted in clinical settings due to their ability to identify tumors at an early stage, which is crucial for improving patient outcomes [5]. However, despite the advancements in imaging technology, challenges persist in the accurate and timely interpretation of breast images. The complexity of breast tissue, particularly in younger women with denser breast tissue, can obscure the visibility of tumors, leading to false negatives [6,7]. Conversely, the sensitivity of these imaging techniques can also result in false positives, leading to unnecessary biopsies and patient anxiety. This dual challenge underscores the need for more sophisticated methods that can enhance the accuracy and efficiency of breast cancer screening.

In recent years, artificial intelligence (AI) has emerged as a transformative tool in the field of medical imaging. AI, particularly using deep learning algorithms, has demonstrated remarkable potential in automating the analysis of medical images, thereby reducing the burden on radiologists and increasing the speed and accuracy of diagnosis. Convolutional neural networks (CNNs), a type of deep learning algorithm, have been particularly successful at image classification tasks, making them well-suited for applications in breast cancer detection [8,9,10,11]. The integration of AI into breast imaging presents several opportunities [12,13]. AI can assist in the interpretation of complex images by highlighting areas of interest that may require further investigation, thus serving as a valuable second opinion for radiologists [14,15,16]. Additionally, AI can enhance the speed of image analysis, enabling quicker decision-making and potentially reducing the time between screening and diagnosis [17,18,19,20].

AI can also improve the consistency of image interpretation, reducing variability caused by human factors, such as fatigue or differences in experience levels among radiologists [21]. Despite these advantages, the application of AI in breast imaging is not without challenges [22,23]. A primary challenge is the need for large, diverse datasets to effectively train AI models [24]. These datasets must encompass a wide range of imaging modalities, including mammography and ultrasound, and must reflect the variability in breast tissue across different populations [25,26]. Furthermore, developing AI models that are able to generalize effectively across different imaging modalities is critical [27]. This necessitates robust training processes and the integration of cross-modality data to ensure that AI systems interpret images accurately, regardless of the imaging technique used.

To address these challenges, this study focuses on the development and application of cross-image and fusion AI methods. The primary goal is to improve the breast cancer recognition speed and screening accuracy by leveraging the strengths of both mammography and ultrasound imaging. By integrating these imaging modalities, this study aims to create a more comprehensive diagnostic tool that provides a richer set of features for AI models to learn from. This approach is expected to enhance the ability of AI to detect tumors, particularly in cases where one imaging modality alone might be insufficient [28,29,30].

## 2. Materials and Methods

The breast images used in this study were sourced from reputable public databases, including the Radiological Society of North America, the Polish Academy of Sciences, and Kaggle. These datasets provide a diverse collection of images, including B-mode ultrasound and mammography images, which are essential for training robust AI models. The images were divided into two categories: malignant and benign tumors. To address the imbalance in the dataset, particularly in the number of ultrasound images, data augmentation techniques were employed. This process involved generating additional samples from the existing images, thereby increasing the dataset’s size and variability and improving the AI model’s ability to generalize across different cases [31,32].

The methodology in this study is structured around three distinct models. The first model involves using CNNs to separately extract features from ultrasound and mammography images, which are then integrated into a machine learning classification model. This approach allows the model to learn from the unique characteristics of each imaging modality, before combining them to make a final classification. The second model takes a more integrated approach by incorporating both ultrasound and mammography sets simultaneously to build a CNN classification model. This model is designed to leverage the complementary strengths of both imaging techniques, providing a more holistic view of the breast tissue. The third model is built on the second by extracting features after the initial CNN classification and using these features to construct a separate machine learning model [33]. This layered approach aims to refine the classification process further and improve the overall accuracy of the model.

**Figure 1:**
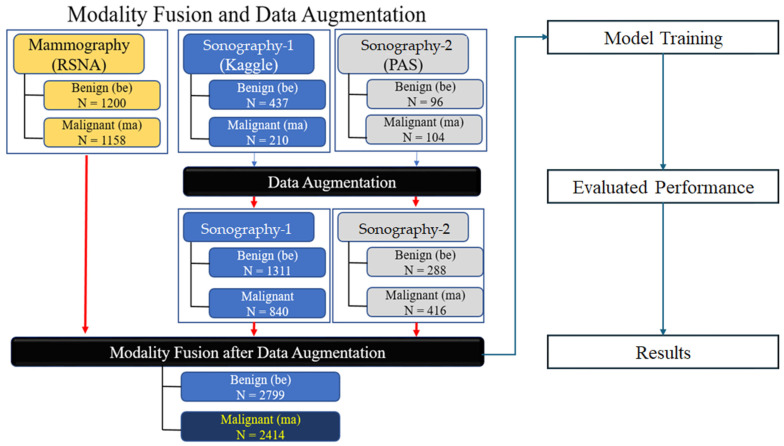
Workflow of modality fusion and data augmentation for breast cancer detection.

Figure 1 shows the flowchart used in this study. The study begins with the input modality fusion after data augmentation, involving 2799 benign and 2414 malignant images after applying data augmentation. The data are then processed through three distinct models: Model 1 involves the use of pre-trained CNNs for feature extraction, followed by machine learning classification; Model 2 utilizes pre-trained CNNs for transfer learning, with all the weights retrained for image classification; and Model 3 is a user-defined CNN model, with a new round of training for image classification. The models are subsequently evaluated through a training schema, validation of the classification performance, and analysis of the results.

### 2.1. Data Source

**Figure 2:**
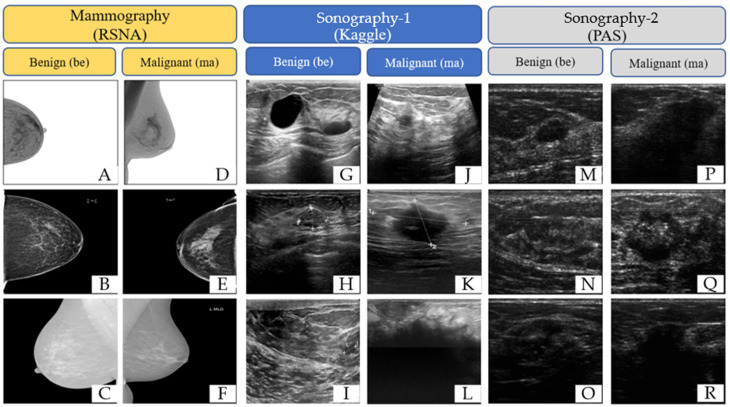
Representative examples of breast imaging modalities used in the study. The mammography (RSNA) for benign (**A**–**C**) and malignant (**D**–**F**) cases, sonography-1 (Kaggle) for benign (**G**–**I**) and malignant (**J**–**L**) cases, and sonography-2 (PAS) for benign (**M**–**O**) and malignant (**P**–**R**) cases.

This study utilized breast images from three prominent public databases: the Radiological Society of North America (RSNA), the Polish Academy of Sciences (PAS), and Kaggle (Table 1). Table 1 provides a summary of the data sources and imaging modalities used in this study and the dataset distribution, both before and after data augmentation. The RSNA dataset contains mammography images, with 1200 benign and 1158 malignant cases, and the images were resized to 256 × 256 pixels. The Kaggle dataset includes B-mode ultrasound images, with 437 benign and 210 malignant cases, expanded using data augmentation to 1311 benign and 840 malignant cases. The image sizes in this dataset vary between 324 × 510 and 719 × 811 pixels. Similarly, the Polish Academy of Sciences dataset comprises B-mode ultrasound images, with 96 benign and 104 malignant cases originally, augmented to 288 benign and 416 malignant cases, with an image size of 256 × 256 pixels. The overall dataset after augmentation consists of 2799 benign and 2414 malignant images. The table illustrates the diversity of the data sources and modalities used in this study and highlights the role of augmentation in addressing dataset imbalances. The diversity of these datasets allowed for the development of AI models capable of performing well across different imaging modalities and patient populations. The images are categorized by modality and diagnosis: mammography (RSNA) for benign (A–C) and malignant (D–F) cases, sonography-1 (Kaggle) for benign (G–I) and malignant (J–L) cases, and sonography-2 (PAS) for benign (M–O) and malignant (P–R) cases. These images illustrate the diversity of the data sources and imaging modalities utilized for the classification tasks in this research (Figure 2).

### 2.2. Data Preprocessing and Augmentation

Before model development, the images underwent several preprocessing steps to ensure consistency and quality. First, the images were resized to a uniform dimension, with both mammography and ultrasound images adjusted to 300 × 300 pixels. The choice to resize the images to 300 × 300 pixels was made to balance computational efficiency with the preservation of important image features. This size ensures that the model can process images efficiently, while retaining sufficient resolution for the CNN to capture key diagnostic features in both mammography and ultrasound images. This standardization was necessary to facilitate the input of images into the CNN models. Additionally, data augmentation techniques were applied to the ultrasound images to address the imbalance between the number of malignant and benign cases. The data augmentation included random rotations (up to ±30 degrees), horizontal and vertical flipping, zooming (range: 0.8–1.2×), and Gaussian noise addition. These parameters were selected to increase the dataset’s diversity, improve model generalizability, and mitigate overfitting. The use of augmentation was particularly critical for the ultrasound dataset, where the number of images was significantly lower compared to the mammography dataset.

### 2.3. Model Development

The study developed three distinct models, each designed to explore different strategies for improving breast cancer detection through AI-driven image analysis. The analysis was conducted using a high-performance workstation, equipped with an Intel Core i9-12900K processor, 64 GB RAM, an NVIDIA RTX 3090 GPU (24 GB DDR5 RAM), and 2 TB SSD storage, ensuring efficient handling of computationally intensive tasks and the data augmentation. The software environment included MATLAB R2024a, with the Deep Learning Toolbox, running on Windows 11.

The selection of the pre-trained CNN models was based on their proven performance in medical imaging tasks and their suitability in terms of the dataset characteristics in this study. The models were chosen due to the following reasons:

1. Image Types and Dataset Characteristics:
  - The dataset includes both mammography and B-mode ultrasound images, which vary significantly in terms of texture, contrast, and spatial resolution. The selected models have been shown to perform well with similar medical imaging datasets, involving high variability in regard to the image features;
2. Prior Performance in Studies:
  - EfficientNetB0 was chosen for its ability to achieve an optimized balance between accuracy and computational efficiency, achieved through compound scaling. Its lightweight architecture is particularly well-suited for medical imaging tasks, where computational resources may be limited;
  - MobileNetV2 was selected for its streamlined architecture, which makes it computationally efficient and capable of handling tasks involving smaller datasets. Its performance in prior studies has demonstrated its robustness in extracting features from ultrasound and mammography images, which have varying textures and contrasts;
  - InceptionV3 was included due to its multi-scale feature extraction capability, achieved through inception modules. This model is particularly effective when used on datasets containing heterogeneous image characteristics, such as the fusion of mammography and ultrasound data in this study;
  - ResNet50 was chosen for its deep residual network architecture, which allows for the effective training of deeper models by addressing vanishing gradient issues. It has been widely adopted for medical imaging tasks, demonstrating superior performance in extracting hierarchical features;
  - ResNet101, as a deeper version of ResNet, was included for its enhanced ability to capture fine-grained details in complex datasets. Its depth makes it particularly suitable for analyzing cross-modality imaging data;
3. Applicability in regard to the Study Objectives:
  - The selected models align well with the need to handle diverse image features from two modalities and to leverage transfer learning to enhance the models’ performance when using a relatively small dataset.

The selection of the three machine learning algorithms, Support Vector Machine (SVM), Logistic Regression (LR), and Naïve Bayes (NB), was based on their complementary characteristics and suitability for different aspects of the classification tasks involving Model 1.

SVM was chosen for its effectiveness in high-dimensional spaces and its ability to handle non-linear relationships using kernel functions. For this study, a radial basis function (RBF) kernel was employed, as it is well-suited to handling non-linear separability in complex datasets. The primary hyperparameters used were:

- C (Regularization Parameter): Set to 1, balancing the trade-off between maximizing the margin and minimizing classification errors;
- Gamma: Set to 1/(number of features), controlling the influence of individual data points on the decision boundary.

LR was selected for its simplicity, interpretability, and suitability for binary classification tasks. The algorithm fits a linear decision boundary and provides probabilistic outputs, which are useful for threshold-based decision-making. The following hyperparameters were used:

- Regularization Type: L2 regularization to prevent overfitting;
- Regularization Strength (C): Set to 1, ensuring a balance between underfitting and overfitting.

NB was included due to its computational efficiency and ability to perform well using small datasets, even under the assumption of feature independence. A Gaussian Naïve Bayes variant was used in this study. The following parameter was used:

- Variance Smoothing Parameter: Set to 1e-9 to stabilize calculations for small datasets and avoid zero probabilities in regard to feature distributions.

#### 2.3.1. Model 1: Fusion of Modalities Using Pre-Trained CNNs

**Figure 3:**
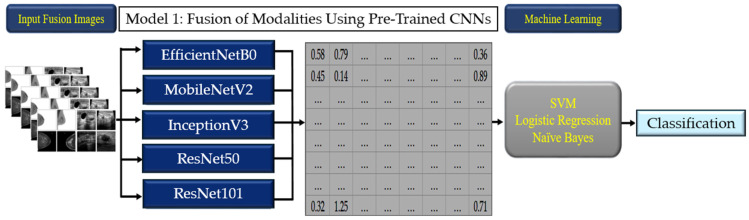
The workflow of Model 1.

The first model involved the use of CNNs to separately extract features from ultrasound and mammography images. The CNNs employed were pre-trained on the ImageNet dataset, allowing them to leverage previously learned features. The extracted features from each modality were then integrated into a machine learning classification model. The machine learning algorithms used for this classification included Logistic Regression (LR), Naïve Bayes (NB), and Support Vector Machines (SVMs). The combination of CNN feature extraction and traditional machine learning classifiers aimed to enhance the interpretability and performance of the model (Figure 3).

#### 2.3.2. Model 2: Fusion of Modalities Using Transfer Learning

**Figure 4:**
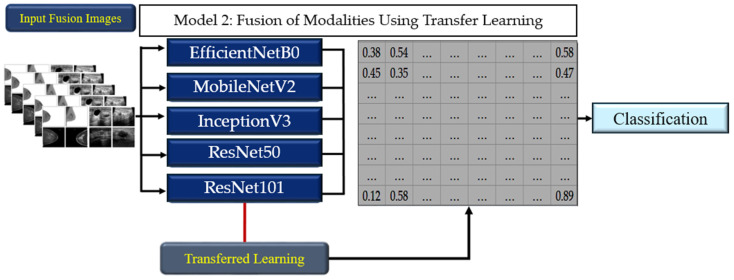
The workflow of Model 2.

The second model took a more integrated approach, by simultaneously incorporating both ultrasound and mammography image sets into the CNN classification models. Model 2 employed a transfer learning approach to fuse the modalities and enhance the classification performance. Pre-trained CNN architectures, including EfficientNetB0, MobileNetV2, InceptionV3, ResNet50, and ResNet101, were retrained in regard to the weights of the fused mammography and ultrasound images. All the input images were resized to 300 × 300 pixels to ensure compatibility with the pre-trained models, while preserving important diagnostic features. The models were trained using Stochastic Gradient Descent with Momentum (SGDM), Adaptive Moment Estimation (ADAM), and Root Mean Square Propagation (RMSprop), each configured with a learning rate of 0.001. Training was conducted over 30 epochs, with a batch size of 30 images per iteration, to ensure efficient convergence, while balancing the computational demands. To prevent overfitting, early stopping was applied, halting training if the validation loss did not improve for 5 consecutive epochs. This approach allowed the models to leverage the pre-trained weights from large-scale image datasets, adapting them to the fused medical imaging data for enhanced feature extraction and classification. The transfer learning strategy effectively utilized the strengths of the pre-trained architecture, providing a robust framework for analyzing the fused modalities (Figure 4).

#### 2.3.3. Model 3: User-Designed CNN Layers for Classification

**Figure 5:**
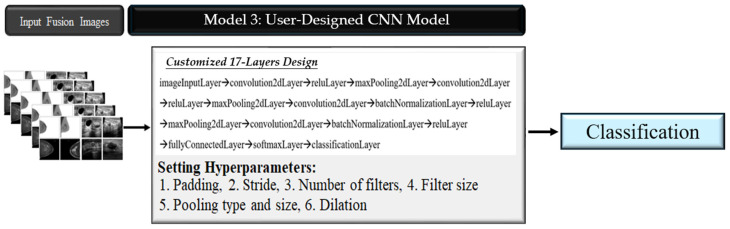
The workflow of Model 3.

This model features a custom 17-layer CNN architecture specifically designed for classifying fused images from both ultrasound and mammography datasets (Figure 5). Key hyperparameters, such as padding, stride, the number of filters, the filter size, pooling type and size, and dilation, were carefully selected to optimize the model’s performance. The rationale for using a 17-layer CNN model has been included in the manuscript. The design choice was based on achieving an optimal balance between model complexity and performance. The 17-layer architecture was specifically tailored to capture the intricate features of fused mammography and ultrasound data, while avoiding overfitting, which could occur with deeper networks. Figure 5 illustrates the detailed design of this architecture, providing additional context on this decision. The hyperparameters for the training models are similar to those used in Model 2, including a learning rate of 0.001 and an early stopping schema. Also, the training was conducted over 25 and 30 epochs, with a batch size of 30 and 64 images per iteration.

#### 2.3.4. Model Training and Testing

An 80/20 split was selected as a widely recognized practice in machine learning, providing an effective balance between training the model and reserving sufficient data for testing. This approach ensures the model has adequate data for learning from, while enabling a robust evaluation using an independent test set to assess the model’s generalizability. The dataset was divided into training and testing sets, with 80% allocated for training and the remaining 20% reserved for testing, across all three models.

### 2.4. Evaluation Metrics

The performance of each model was evaluated using a set of standard classification metrics. These included accuracy, specificity, sensitivity, positive predictive value (PPV), negative predictive value (NPV), and the Kappa statistic. Accuracy measured the overall correctness of the model’s predictions, while specificity and sensitivity provided insights into the model’s ability to correctly identify benign and malignant cases, respectively. The PPV and NPV offered additional context by evaluating the proportion of true positive and true negative results among all the positive and negative predictions. The Kappa statistic was used to measure the agreement between the predicted and actual classifications, accounting for the possibility of agreement occurring by chance. The ROC curve was applied to investigate the performance of the models.

## 3. Results

### 3.1. Model 1: Fusion Modalities Using Pre-Trained CNNs with Classifiers

In regard to the first model, pre-trained CNNs were used to extract the features from both the B-mode ultrasound and mammography images. The extracted features were then fed into traditional machine learning classifiers, including LR, NB, and SVM. The combined use of CNN feature extraction and machine learning classifiers yielded moderate classification performance (Table 2). The highest accuracy was achieved by the EfficientNetB0 CNN combined with the SVM classifier, resulting in an accuracy of 0.769 and a Kappa score of 0.534. The sensitivity and specificity were 0.781 and 0.754, respectively. Other pre-trained models, such as InceptionV3, MobileNetV2, ResNet50, and ResNet101, showed varying levels of performance, but none surpassed the results obtained with EfficientNetB0 and SVM.

This model demonstrated that integrating pre-trained CNNs with traditional classifiers can provide a decent starting point for breast cancer classification, although the performance was limited by the feature extraction capabilities of the CNNs and the generalization capacity of the machine learning classifiers.

**Figure 6:**
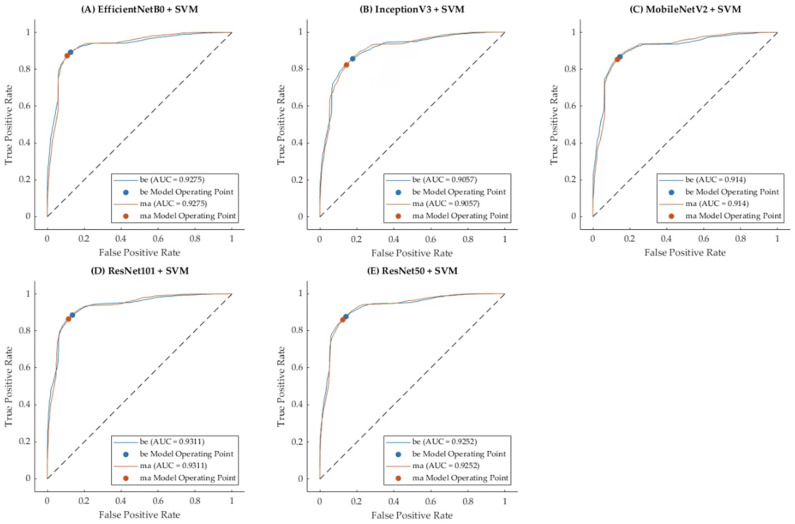
ROC curves for Model 1, with maximum accuracy across classifiers (SVM, LR, and NB) for each pre-trained CNN.

Figure 6 illustrates the Receiver Operating Characteristic (ROC) curves for Model 1, which integrated pre-trained CNN architectures with the SVM classifier, that achieved the highest accuracy compared to Logistic Regression (LR) and Naïve Bayes (NB). The plots display the performance of each pre-trained CNN (EfficientNetB0, InceptionV3, MobileNetV2, ResNet101, and ResNet50) in terms of their true positive rate (sensitivity) and false positive rate (specificity) across various thresholds. The Area Under the Curve (AUC) values, denoted on each plot, provide a quantitative measure of the classification performance. EfficientNetB0 and ResNet101 achieved the highest AUC values of 0.9275 and 0.9311, respectively, indicating superior discriminative capability in classifying benign (be) and malignant (ma) cases. These ROC curves highlight the robustness of Model 1 when paired with SVM, effectively leveraging the strengths of different CNN architectures to handle cross-modality fused input data.

### 3.2. Model 2: Fusion Modalities Using Transfer Learning with Classifiers

The second model explored the use of transfer learning by applying pre-trained CNNs to fusion modalities (Table 3). The features extracted from the CNNs were then fused and fed into a single classification model. Transfer learning significantly improved the classification performance compared to Model 1. The best results were observed with the EfficientNetB0 model using the SGDM optimizer. This combination achieved an accuracy of 0.820 and a Kappa score of 0.644. The application of transfer learning allowed the models to adapt better to the specific characteristics of the breast imaging datasets, leveraging the knowledge from pre-training using a large, diverse dataset.

The integration of transfer learning with multiple imaging modalities resulted in a more robust model, better equipped to handle the complex task of breast cancer classification. This approach outperformed the initial method, underscoring the effectiveness of transfer learning in medical image classification.

**Figure 7:**
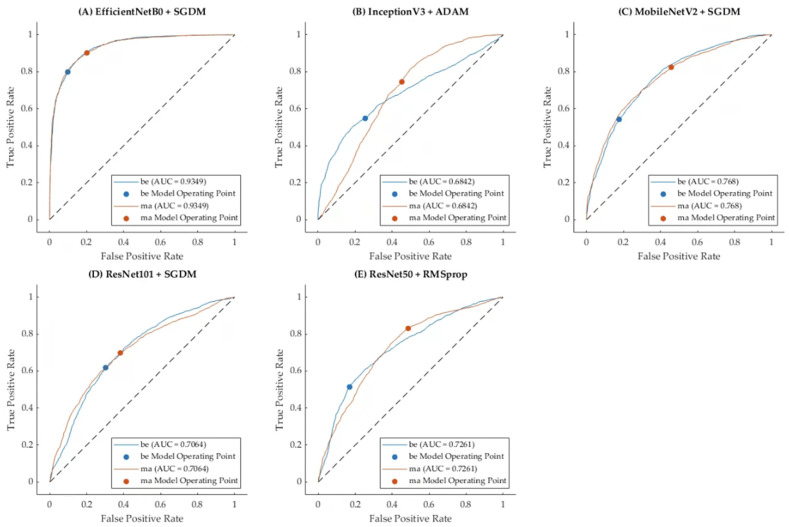
ROC curves for Model 2, with maximum accuracy across optimizers (ADAM, RMSprop, and SGDM) for each transfer learning CNN.

Figure 7 presents the Receiver Operating Characteristic (ROC) curves for Model 2, which employed transfer learning-based CNN architectures combined with three optimization algorithms, namely ADAM, RMSprop, and SGDM for classified benign (be) and malignant (ma) cases. The plots display the ROC curves for each pre-trained CNN (EfficientNetB0, InceptionV3, MobileNetV2, ResNet101, and ResNet50) using the optimizer that achieved the highest accuracy. The Area Under the Curve (AUC) values, annotated on each plot, quantify the discriminative performance of the models. EfficientNetB0 with SGDM achieved the highest AUC (0.9349), showcasing its superior ability to distinguish between benign and malignant cases. In contrast, InceptionV3 with ADAM resulted in the lowest AUC (0.6842), reflecting lower classification effectiveness. These results underscore the importance of choosing the most appropriate optimizer when fine-tuning transfer learning models and highlight SGDM’s robustness in achieving high sensitivity and specificity in breast cancer detection, using fused mammography and ultrasound images.

### 3.3. Model 3: User-Designed CNN Layers for Classification

The third model involved the development of a custom-designed 17-layer CNN, specifically tailored for the classification of breast images (Table 4). This user-designed CNN outperformed both Model 1 and Model 2, achieving an accuracy of 0.964 and a Kappa score of 0.927, along with impressive sensitivity (0.976) and specificity (0.964). These results remained consistent across different image sizes and training epochs. The model demonstrated excellent classification performance, with high sensitivity and a high positive predictive value (PPV) for both malignant and benign categories. Specifically, 25 and 30 epochs were selected based on empirical observations to ensure model convergence without overtraining, while batch sizes of 25 and 30 images was chosen to balance computational efficiency and memory usage, given the hardware limitations and dataset size.

**Figure 8:**
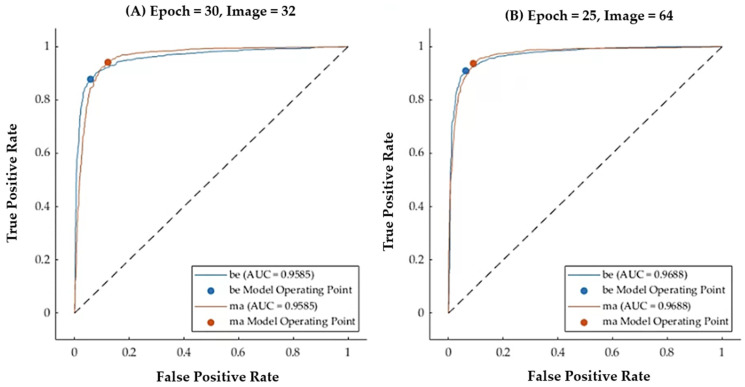
ROC curves for Model 3 (custom CNN), with optimal accuracy based on epochs and batch sizes.

Figure 8 illustrates the Receiver Operating Characteristic (ROC) curves for Model 3 (user-designed CNN) for different training configurations, showcasing the highest accuracy achieved based on varying epochs and batch sizes. Subfigure (A) represents the model’s performance with 30 epochs and a batch size of 32 images, achieving an AUC of 0.9585 for both benign (be) and malignant (ma) cases. Subfigure (B) shows the model’s performance with 25 epochs and a batch size of 64 images, achieving a slightly higher AUC of 0.9688 for both classes of images. These results highlight the impact of training in regard to the hyperparameters on the model’s ability to discriminate between benign and malignant cases. The configuration in (B) demonstrates superior discriminative power, suggesting that a slightly lower number of epochs combined with a larger batch size optimizes the balance between computational efficiency and model performance.

The superior performance of this model underscores the effectiveness of the custom-designed CNN architecture for this application. The ability to fine-tune each layer and optimize the network to account for the nuances in breast imaging data proved to be highly advantageous, resulting in the best performance among the three models.

The findings suggest that for clinical applications where accuracy and reliability are paramount, a custom-designed CNN, such as Model 3, would be the most appropriate choice. The other models, particularly Model 2, could serve as effective alternatives in settings where computational resources are more limited, but the need for a high level of accuracy remains critical.

The results of this study highlight the potential of AI-driven methods in breast cancer screening. The pre-trained CNN combined with machine learning demonstrated solid classification performance, with notable improvements observed when using transfer learning techniques. The user-designed CNN, a 17-layer model specifically tailored for this study, achieved the highest accuracy and Kappa score among the models tested, indicating its effectiveness in classifying breast images. These findings suggest that the integration of cross-modality data and AI fusion techniques can significantly enhance the detection and diagnosis of breast cancer.

The application of AI in breast cancer screening holds great promise for improving the accuracy and efficiency of diagnosis. By leveraging the strengths of multiple imaging modalities and advanced AI techniques, this study contributes to the ongoing efforts to develop more effective tools for breast cancer detection. The results underscore the importance of continued research and development in this area, particularly in the creation of AI models that can generalize effectively across different imaging modalities and patient populations. As the field of AI in medical imaging continues to evolve, it is expected that these technologies will play an increasingly vital role in the fight against breast cancer.

## 4. Discussion

The findings from this study highlight the significant potential of advanced AI techniques, particularly CNNs, in enhancing the accuracy and efficiency of breast cancer detection through medical imaging. By comparing three distinct models, each employing different strategies for feature extraction, classification, and model design, this research provides valuable insights into the strengths and limitations of various AI-driven approaches for breast cancer classification.

### 4.1. Model Performance and Implications

The user-designed 17-layer CNN (Model 3) demonstrated superior performance compared to the other two models, achieving an accuracy of 0.964 and a Kappa score of 0.927. This high level of accuracy indicates that a custom-designed CNN architecture, specifically tailored to the task, can effectively capture the complex patterns and features present in breast imaging data. The model’s ability to consistently distinguish between malignant and benign tumors suggests that it could be a powerful tool in clinical settings, potentially reducing the incidence of both false positives and false negatives. In contrast, the pre-trained CNNs combined with traditional machine learning classifiers (Model 1) showed the lowest performance, with an accuracy of 0.780 and a Kappa score of 0.559. While this approach provided a solid baseline, the results highlight the limitations of relying solely on pre-trained networks without further adaptation to the specific dataset. The relatively modest performance underscores the importance of developing models that are finely tuned to the characteristics of the target data, especially in complex tasks like medical image classification.

The fusion of modalities through transfer learning (Model 2) provided a significant improvement over Model 1, achieving an accuracy of 0.846 and a Kappa score of 0.694. This model demonstrated the benefits of leveraging transfer learning, which allowed the CNNs to apply learned features from a large, diverse dataset to the specific task of breast cancer classification. The improvement in performance suggests that transfer learning is a valuable strategy, particularly when working with limited labeled data, as it enables the model to benefit from previously acquired knowledge.

Table 5 provides comparative analysis of the computational metrics for various models, including the executed inference time, parameter size, and the number of layers. Among the pre-trained models combined with SVM, EfficientNetB0 had the longest execution time (79.0 s) due to its larger architecture, while MobileNetV2 demonstrated the shortest time (27.2 s), reflecting its lightweight design. The custom-designed CNN model achieved significantly reduced execution times, with 4.9 s for 30 epochs and a batch size of 32 images and 6.7 s for 25 epochs and a batch size of 64 images. Additionally, the parameter sizes and layer counts highlight the efficiency of the user-designed CNN compared to pre-trained architectures, with parameter sizes of 678.9 MB and 679.6 MB, and a consistent layer count of 17. These results suggest that the custom-designed CNN not only improves the classification performance, but also minimizes computational demands, making it a practical solution for real-time clinical applications. The findings underscore the importance of optimizing both the model architecture and the hyperparameters to achieve a balance between performance and computational efficiency.

### 4.2. Comparison with Similar Studies in the Literature

Table 6 provides comparative analysis of the methods in regard to similar studies in the literature, highlighting differences in the data sources, sample sizes, methodologies, and accuracy rates. Previous studies, such as those by Byra M et al. (2019) and AlZoubi A et al. (2024), employed transfer learning and DCNN approaches, using US-based datasets, achieving accuracy rates of 0.887 and 0.894, respectively. Jabeen K et al. (2022) reported a high accuracy of 0.993 using a probability-based serial approach, while Pesapane F et al. (2023) and Jaamour A et al. (2023) utilized CNNs and mammography data, achieving accuracy rates of 0.950 and 0.674, respectively. Michael E et al. (2024) demonstrated near-perfect accuracy (0.999) using CNNs on mammography data [25,34,35,36,37,38].

In comparison, the proposed methods, utilizing a custom-designed CNN, achieved an accuracy of 0.923 for mammography data and 0.943 for fused modalities. The relatively larger sample size (3205 for fusion modalities) and the integration of cross-modality imaging contributed to the improved performance, indicating the potential of fusion-based approaches for enhanced breast cancer detection. These results demonstrate the effectiveness of the proposed methods and their competitive performance compared to state-of-the-art techniques in literature.

### 4.3. The Role of Cross-Modality Imaging

**Figure 9:**
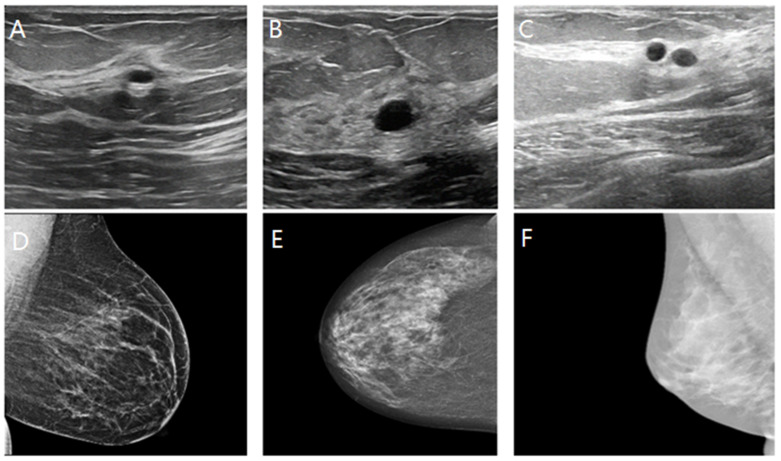
Comparison of breast tissue textures in sonography and mammography images.

A key contribution of this study is the successful integration of cross-modality imaging, which combines mammography and ultrasound images, to enhance classification accuracy. By fusing features from these different imaging modalities, the models were able to leverage the complementary strengths of each, resulting in more robust and accurate classifications. For example, mammography excels at visualizing dense structures, while ultrasound provides detailed images of soft tissues. This integration allowed the AI models to perform more comprehensive analysis of breast tissue, which is crucial for accurate tumor detection. Ultrasound images, as shown in Figure 9A–C, depict various breast tissue characteristics, while mammography images, as shown in Figure 9D–F, illustrate different breast tissue densities. These images were instrumental in training and testing the AI models for breast cancer detection and classification.

The findings suggest that cross-modality imaging can significantly enhance AI-driven diagnostic tools, making them more reliable and versatile in different clinical scenarios. This approach is particularly beneficial in cases where a single imaging modality may not provide sufficient information for an accurate diagnosis. The ability to combine and analyze data from multiple sources could lead to earlier detection and better treatment outcomes for patients with breast cancer.

### 4.4. Clinical Implications

The findings of this study have significant clinical implications for breast cancer screening and diagnosis. The integration of mammography and ultrasound imaging using a custom-designed 17-layer CNN demonstrated superior diagnostic accuracy, sensitivity, and specificity, which could facilitate earlier and more reliable breast cancer detection. By leveraging the complementary strengths of these imaging modalities, the proposed approach addresses limitations such as decreased sensitivity in dense breast tissue and false positives in mammography. This has the potential to reduce diagnostic variability and improve the consistency of interpretations across radiologists, ultimately leading to more effective clinical workflows. Furthermore, the computational efficiency of the custom model makes it feasible for real-time deployment in clinical settings, offering healthcare professionals a robust and reliable diagnostic aid. As the method is further developed and validated with larger and more diverse datasets, it could serve as a critical tool for improving patient outcomes through timely and accurate diagnosis, particularly in resource-limited environments.

### 4.5. Limitations and Future Directions

**Figure 10:**
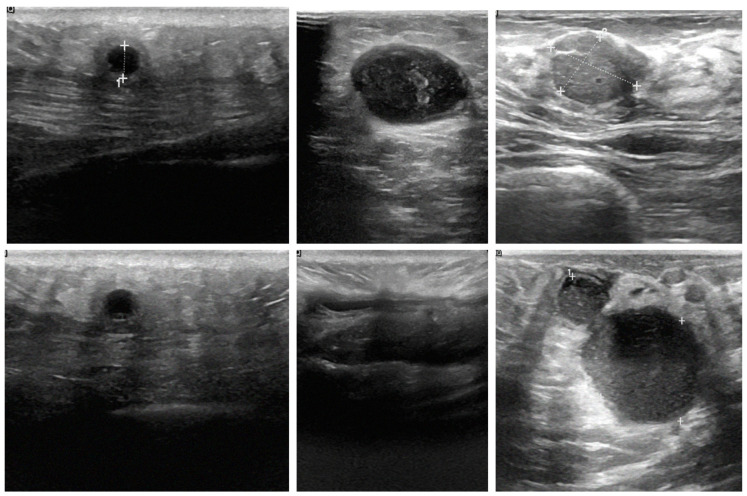
Examples of varying resolutions in breast sonography images.

**Figure 11:**
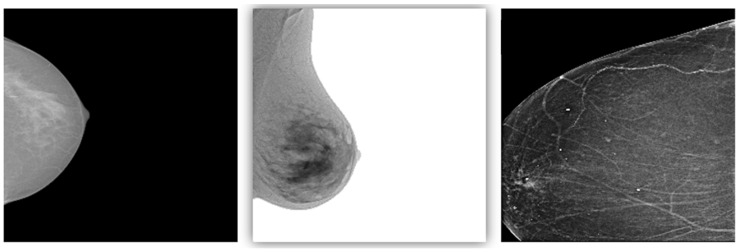
Examples of varying contrast levels in mammography images.

Despite the promising results, this study has several limitations that should be addressed in future research. First, the study relied on public datasets, which, although diverse, may not fully capture the variability encountered in clinical practice (Figure 10 and Figure 11). The datasets used in this study were relatively balanced after augmentation; however, real-world clinical data often exhibit significant imbalances, particularly between malignant and benign cases. To ensure that models generalize effectively across different populations, future studies should incorporate larger and more diverse datasets that encompass a broader range of patient demographics and imaging conditions.

Second, the computational complexity of the user-designed CNN (Model 3) may limit its practicality in resource-constrained settings. While the model’s superior performance justifies its use in high-stakes clinical environments, further work is needed to optimize the model’s efficiency without compromising accuracy. Techniques such as model pruning, quantization, or the development of lightweight architectures could be explored to reduce the computational demands of the model. Specifically, the model’s demands included extended training times, increased memory usage due to its deeper architecture, and the need for a high level of computational resources during both training and inference stages.

Third, while the study demonstrated the potential of AI in improving breast cancer detection, the interpretability of AI models remains a critical challenge. In clinical practice, it is essential that AI-driven decisions can be explained and understood by medical professionals. Future research should focus on developing explainable AI models that not only provide accurate predictions, but also offer insights into the decision-making process. This could involve the use of attention mechanisms, feature importance maps, or other techniques that help to visualize and interpret the features contributing to the model’s decisions. Lastly, the studies mentioned in this article offer valuable insights into the potential benefits of utilizing datasets where both imaging modalities are available for the same subjects. The referenced studies will be included in the manuscript, along with a discussion on how such datasets could facilitate a more comprehensive evaluation of the custom-designed 17-layer CNN model. Incorporating dual-modality data or 3D breast ultrasound images could further enhance the model’s performance by increasing the depth and richness of the fused features [39,40].

### 4.6. Clinical Impact and Future Applications

The results of this study underscore the potential for AI-driven models to significantly enhance breast cancer screening and diagnosis. By improving the accuracy and speed of image analysis, these models could reduce the workload on radiologists, allowing them to focus on more complex cases. The integration of cross-modality imaging, as demonstrated in this research, could become the standard approach in regard to the future use of diagnostic tools, providing a more comprehensive assessment of breast health.

Looking ahead, the methodologies and findings from this study could be extended to other areas of medical imaging. For example, similar AI-driven approaches could be applied to the detection of other cancers or diseases where multiple imaging modalities are used. Furthermore, the development of AI models that can integrate data from non-imaging sources, such as patient history or genetic information, could lead to even more personalized and accurate diagnostic tools.

## 5. Conclusions

This study demonstrates the significant potential of advanced AI techniques, particularly convolutional neural networks (CNNs), in enhancing breast cancer detection through the integration of cross-modality imaging. The study developed and evaluated three distinct models, each employing unique approaches to feature extraction, classification, and design. Among these, the custom-designed 17-layer CNN (Model 3) emerged as the most effective, achieving an accuracy of 0.964 and a Kappa score of 0.927. This superior performance highlights the value of tailoring the AI architecture to the specific characteristics of the imaging data and the diagnostic tasks.

The integration of mammography and ultrasound images proved to be highly beneficial, enabling more comprehensive analysis that enhanced screening accuracy. The cross-modality fusion approach leverages the complementary strengths of both imaging modalities, contributing to more reliable and earlier breast cancer detection. This advancement has the potential to significantly improve patient outcomes by facilitating timely diagnosis and treatment.

The successful integration of multiple imaging modalities underscores the feasibility and potential of combining diverse datasets for AI-driven diagnostic tools. However, the study also highlights the need for larger and more diverse datasets to improve model generalizability across populations. Future research should focus on optimizing computational efficiency, incorporating explainable AI techniques, and collaborating with local medical institutions to include population-specific imaging data, such as Taiwanese datasets.

Finally, the proposed approach offers significant benefits for healthcare personnel by improving diagnostic accuracy, reducing variability, and supporting radiologists in regard to their workflow. This study lays a strong foundation for future research and clinical applications, with the goal of enhancing breast cancer detection and patient care through innovative AI-driven solutions.

## Figures and Tables

**Table 1:** Summary of data sources, imaging modalities, and dataset distribution before and after augmentation.

Data Source	Modality	Original		Augmentation		Image Size
		Benign	Malignant	Benign	Malignant	
RSNA	Mammography	1200	1158	1200	1158	256 × 256
Kaggle	B-mode ultrasound	437	210	1311	840	324 × 510~719 × 811
Polish Academy of Sciences	B-mode ultrasound	96	104	288	416	256 × 256
Total		1733	1472	2799	2414	

Note: Data sources include the RSNA Breast Cancer Detection Dataset (https://www.kaggle.com/datasets/gauravduttakiit/mammography-breast-cancer-detection, accessed on 28 June 2024), the Polish Academy of Sciences Dataset (http://bluebox.ippt.gov.pl/~hpiotrzk, accessed on 2 July 2024), and the Kaggle Breast Ultrasound Images Dataset (https://www.kaggle.com/datasets/aryashah2k/breast-ultrasound-images-dataset, accessed on 4 July 2024).

**Table 2:** Performance metrics of pre-trained CNN models combined with machine learning classifiers.

Model 1	Sensitivity	Specificity	PPV	NPV	Accuracy	Kappa
EfficientNetB0 + LR	0.783	0.676	0.665	0.768	0.713	0.428
EfficientNetB0 + NB	0.648	0.561	0.564	0.645	0.601	0.207
EfficientNetB0 + SVM	0.781	0.754	0.790	0.743	0.769	0.534
InceptionV3 + LR	0.700	0.632	0.662	0.667	0.664	0.328
InceptionV3 + NB	0.691	0.585	0.569	0.705	0.632	0.270
InceptionV3 + SVM	0.742	0.707	0.751	0.697	0.726	0.449
MobileNetV2 + LR	0.639	0.742	0.870	0.429	0.666	0.308
MobileNetV2 + NB	0.717	0.587	0.543	0.752	0.640	0.289
MobileNetV2 + SVM	0.762	0.722	0.760	0.725	0.743	0.484
ResNet101 + LR	0.730	0.679	0.655	0.627	0.642	0.284
ResNet101 + NB	0.649	0.550	0.525	0.671	0.593	0.194
ResNet101 + SVM	0.771	0.726	0.759	0.738	0.749	0.497
ResNet50 + LR	0.790	0.657	0.602	0.746	0.669	0.344
ResNet50 + NB	0.679	0.570	0.544	0.701	0.617	0.241
ResNet50 + SVM	0.763	0.721	0.758	0.727	0.743	0.484

**Table 3:** Performance metrics of transfer learning by applying pre-trained CNNs to fusion modalities.

Model 2	Sensitivity	Specificity	PPV	NPV	Accuracy	Kappa
EfficientNetB0 + ADAM	0.803	0.596	0.496	0.826	0.649	0.314
EfficientNetB0 + RMSprop	0.777	0.644	0.616	0.781	0.692	0.390
EfficientNetB0 + SGDM	0.906	0.754	0.742	0.911	0.820	0.644
InceptionV3 + ADAM	0.681	0.566	0.530	0.711	0.614	0.236
InceptionV3 + RMSprop	0.537	0.463	0.667	0.333	0.512	0.010
InceptionV3 + SGDM	0.633	0.649	0.740	0.462	0.611	0.205
MobileNetV2 + ADAM	0.832	0.574	0.414	0.899	0.639	0.302
MobileNetV2 + RMSprop	0.869	0.582	0.423	0.916	0.651	0.327
MobileNetV2 + SGDM	0.764	0.632	0.606	0.742	0.669	0.343
ResNet101 + ADAM	0.603	0.535	0.653	0.471	0.568	0.122
ResNet101 + RMSprop	0.517	0.305	0.808	0.142	0.499	0.020
ResNet101 + SGDM	0.678	0.573	0.546	0.701	0.618	0.243
ResNet50 + ADAM	0.757	0.560	0.433	0.831	0.618	0.256
ResNet50 + RMSprop	0.789	0.560	0.409	0.872	0.623	0.271
ResNet50 + SGDM	0.709	0.559	0.463	0.784	0.612	0.240

**Table 4:** Performance metrics of the user-designed CNN model across different epochs and batch sizes.

Epoch	Image	Sensitivity	Specificity	PPV	NPV	Accuracy	Kappa
25	32	0.976	0.946	0.954	0.976	0.946	0.890
	64	0.976	0.964	0.970	0.973	0.964	0.927
30	32	0.988	0.979	0.984	0.988	0.957	0.915
	64	0.972	0.971	0.976	0.968	0.962	0.925

**Table 5:** Comparison of computational metrics across models, including executed time, parameter size, and number of layers in the model.

Model	Executed Time (s)	Parameter Size (MB)	Number of Layers
EfficientNetB0 + SVM	79.0	77.4	290
InceptionV3 + SVM	37.9	92.1	315
MobileNetV2 + SVM	27.2	14.2	154
ResNet101 + SVM	41.2	173.0	347
ResNet50 + SVM	27.4	99.7	177
EfficientNetB0 + SGDM	25.9	113.9	290
InceptionV3 + ADAM	11.3	84.2	315
MobileNetV2 + SGDM	15.0	9.2	154
ResNet101 + SGDM	17.4	163.9	347
ResNet50 + RMSprop	12.0	90.6	177
User-Designed CNN: Epochs (30) and Images (32)	4.9	678.9	17
User-Designed CNN: Epochs (25) and Images (64)	6.7	679.6	17

**Table 6:** Comparison of the proposed methods with similar studies in literature.

Authors	Years	Data	Sample Size	Methods	Accuracy
Jabeen K et al. [25]	2022	Breast Ultrasound	780	Probability-based serial approach	0.993
Byra M et al. [34]	2019	Breast Ultrasound	882	Transfer learning	0.887
AlZoubi A et al. [35]	2024	Breast Ultrasound	1289	DCNN	0.894
Pesapane F et al. [36]	2023	Mammography	1000	CNN	0.950
Jaamour A et al. [37]	2023	Mammography	10239	CNN	0.674
Michael E et al. [38]	2024	Mammography	322	CNN	0.999
The proposed methods	2024	Breast Ultrasound	847	User-designed CNN	0.998
		Mammography	2358		0.923
		Fusion Modalities	3205		0.943

## Data Availability

The data supporting the reported results were obtained from publicly available datasets. The breast imaging data used in this study are available from the following sources: RSNA (Radiological Society of North America), PAS (Polish Academy of Sciences), and Kaggle (https://www.kaggle.com/). Specific dataset links and details can be provided upon request. The datasets used in this study are as follows: RSNA Breast Cancer Detection Dataset (https://www.kaggle.com/datasets/gauravduttakiit/mammography-breast-cancer-detection, accessed on 28 June 2024); Polish Academy of Sciences (PAS) Dataset (http://bluebox.ippt.gov.pl/~hpiotrzk, accessed on 2 July 2024); Kaggle Breast Ultrasound Dataset (https://www.kaggle.com/datasets/aryashah2k/breast-ultrasound-images-dataset, accessed on 4 July 2024). These datasets are publicly accessible, and no direct interaction with human participants was involved in this study.
